# Chronic post-traumatic neuropathic pain of brachial plexus and upper limb: a new technique of peripheral nerve stimulation

**DOI:** 10.1007/s10143-014-0523-0

**Published:** 2014-02-21

**Authors:** Giorgio Stevanato, Grazia Devigili, Roberto Eleopra, Pietro Fontana, Christian Lettieri, Chiara Baracco, Franco Guida, Sara Rinaldo, Marzio Bevilacqua

**Affiliations:** 1 Neurosurgery Unit, Neuroscience Department, “Ospedale dell’Angelo”, General Hospital, Mestre-Venice, Italy; 2 Neurological Unit, Neuroscience Department, University Hospital “S. Maria della Misericordia”, Udine, Italy; 3 Pain Medicine, “Ospedale dell’Angelo”, General Hospital, Mestre-Venice, Italy; 4 Pain Management Unit, Anesthesia Department, “Ospedale S. Maria di Ca’ Foncello”, General Hospital, Treviso, Italy

**Keywords:** Peripheral nerve stimulation (PNS), Traumatic brachial plexus injuries, Neuropathic pain, Quantitative sensory testing (QST)

## Abstract

The aim of the study was to evaluate the effect on pain relief in patients with peripheral neuropathic pain after brachial plexus injuries using an implanted peripheral nerve stimulator applied directly to the nerve branch involved into the axillary cavity. Seven patients with post-traumatic brachial plexus lesions or distal peripheral nerve complaining of severe intractable chronic pain were enrolled in a single-centre, open-label trial. Conventional drugs and traditional surgical treatment were not effective. Patients underwent careful neurological evaluation, pain questionnaires and quantitative sensory testing (QST). Surgical treatment consists of a new surgical technique: a quadripolar electrode lead was placed directly on the sensory peripheral branch of the main nerve involved, proximally to the site of lesion, into the axillary cavity. To assess the effect, we performed a complete neuroalgological evaluation and QST battery after 1 week and again after 1, 6 and 12 weeks. All patients at baseline experienced severe pain with severe positive phenomena in the median (5) and/or radial (2) territory. After turning on the neuro-stimulator system, all patients experienced pain relief within a few minutes (>75 % and >95 % in most), with long-lasting pain relief with a reduction in mean Numerical Rating Scale (NRS) of 76.2 % after 6 months and of 71.5 % after 12 months. No significant adverse events occurred. We recommend and encourage this surgical technique for safety reasons; complications such as dislocation of electrocatheters are avoided. The peripheral nerve stimulation is effective and in severe neuropathic pain after post-traumatic nerve injuries of the upper limbs.

## Introduction

Chronic peripheral neuropathic pain due to peripheral nerve injury often results in significant suffering and impaired quality of life. It is poorly responsive to drugs usually used for the treatment of neuropathic pain (NP) such as anticonvulsants and membrane stabilizers and opioids, even at high dosages [25]. For patients who have no response to medications and high rate of deafferentation (i.e. root avulsion), the ablative dorsal root entry zone (DREZ) lesion procedure is traditionally considered, while spinal cord stimulation (SCS) has been reported as a conservative technique in particular in case of prevalent central mechanisms like in CRPS or root avulsion [14, 18].

Therefore, an effective treatment for neuropathic pain still remains a major clinical challenge. Peripheral nerve stimulation (PNS) has been used by a small group of neurosurgeons for the treatment of chronic peripheral neuropathic pain since 1967 [30]. Although in literature, PNS was demonstrated to be quite successful in the short and medium term [4, 6, 9, 21, 27, 29], PNS has never become a standard technique for treatment of NP syndromes. This may be due to technical difficulties with paddle-type electrodes inserted around the peripheral nerve and to the risk of infections or electrode dislocation.

The mechanism by which PNS produces analgesia is still unclear. One of the possible mechanisms is based on the assumption that direct application of low-intensity, high-frequency electrical current onto a peripheral nerve can elicit the A-beta myelinated fibres and produce analgesia according to the “gate-control” mechanism [16]. Another mechanism proposed was the ortho- and antidromic collision in Aβ fibres [10, 20]. Both mechanisms as a key part of PNS are closely related to the brain function and brain plasticity in particular [22, 26].

Thus, we hypothesized that the implantation of PNS leads directly to the nerve branch mainly involved in the painful syndrome, proximally to the site of injury, at the brachial plexus may produce analgesia or significant pain relief in patients with traumatic nerve injuries. For this purpose, a pathway that is at least partially preserved is required.

In this study, we assessed the short- and medium-term outcomes of PNS in a number of patients with peripheral post-traumatic neuropathic pain in the upper arm by using a new and innovative surgical technique. The aim of the study was to evaluate pain relief in this carefully selected group of patients due the direct stimulation of the nerve branches administered by means of lead inserted with an original surgical technique.

## Methods

### Patients

Patients affected by intractable pain due to peripheral nerve injuries and referred to our neurological unit for advanced pain treatment during the years 2007–2010 were considered for PNS. We selected only patients with sensory sensation preserved in the painful skin area. Patients with root avulsion or spinal cord lesions were excluded.

A detailed medical history and neurological examination were collected. Peripheral neuropathic pain was defined as chronic pain in an area of sensory abnormality corresponding to the nerve lesion and with an onset less than 6 months after the lesion, according to the grading system proposed by the NeuPSIG guideline [7, 28].

We decided to include patients with pain defined as “intractable” for no response to the pharmacological and traditional surgical treatment and “chronic” if present from at least 1 year without significant changes in intensity and characteristics. None of the patients underwent microsurgical DREZotomy.

Patients were carefully selected for PNS after an extensive baseline assessment. Therefore, we identify the following criteria for inclusion: (1) clear identification of an isolated injured nerve (i.e. selective branch of brachial plexus, median nerve, radial nerve) by means of clinical and electroneurographic and electromyographic evaluation as the unique cause of pain; (2) complete although transient pain relief following a diagnostic nerve block with local anaesthetics; (4) poor response (<50 % of pain relief) to all other treatments after a trial period of at least 1 year, including medication use and surgical treatment of the injury site such as neurolytic procedures, neuroma resection, nerve grafting and transection; (4) absence of major psychiatric disorders, such as personality disorders or major depression, as assessed by psychological examination using Minnesota Multiphasic Personality Inventory (MMPI-2); (5) signing of a written informed consent for the study and for the surgical procedure.

The study was single centre and open label and received institutional review board approval.

### Neurological and algological baseline examination

All subjects underwent a complete neurological and algological examination of the upper and lower trunk, medial, lateral and posterior cords of the brachial plexus and peripheral nerves, in order to assess the motor and sensory loss distribution and positive phenomena described below.

Superficial and pinprick sensations were examined in order to identify negative sensory signs (sensory loss) and positive sensory signs (evoked and spontaneous pain, paraesthesias). The neurological examination was performed using cotton gauze (light touch and dynamic mechanic allodynia test) and a brush, disposable safety needle (hypoalgesia, pinprick hyperalgesia, after-sensation test), repetitive pinprick (2 Hz for 30 s) and glass vials filled with cold and hot water (thermal sensation, allodynia test, after-sensation test). Deep tendon reflexes were classified as normal, decreased (if present with reinforcement) or absent. All muscle groups of the upper arm were evaluated, and muscle strength was graded using the Medical Research Council (MRC) score. Intensity of spontaneous pain, allodynia including static mechanical (pressure), dynamic mechanical (brush), heat or cold (thermal) and hyperalgesia were graded using the Numerical Rating Scale (NRS) (0–10) scale. We used the following descriptors for quality of pain: throbbing, lancinating, unpredictable, lightning-like, sharp, shooting, aching, burning, scalding, pruritic [5, 7]. Pain attacks were described in terms of intensity, duration and frequency.

We collected a picture for the pain on the skin and a detailed pain map and sensory abnormality distribution on a body chart.

Finally, the clinical evaluation was completed with upper arm examination in order to rule out any intrinsic joint disease and acromioclavicular pathology, and in patients with lower brachial plexus involvement the elevated arm stress test [20, 23] was performed in order to relieve thoracic outlet syndrome [24].

### Quantitative sensory testing

Thresholds for cold and warm sensation (CS, WS) and cold and heat pain (CP, HP) were obtained bilaterally on the thenar and hypothenar eminences and on other sites according with the neuroanatomical distribution of pain and negative signs, with a 30 × 30 mm thermode of the TSA-II Neuro Sensory Analyzer (Medoc, Israel). Method of limits has been performed. Each test was repeated four times for each side, and the perceived threshold was defined as the average of peak temperatures. Subjects were instructed to push the response button, which recorded the temperature and reset the thermode back to baseline, as soon as they detected a change in temperature for CS and WS, or only if the sensation changed from cold or hot to painful sensation.

## Surgical procedure

**Fig. 1:**
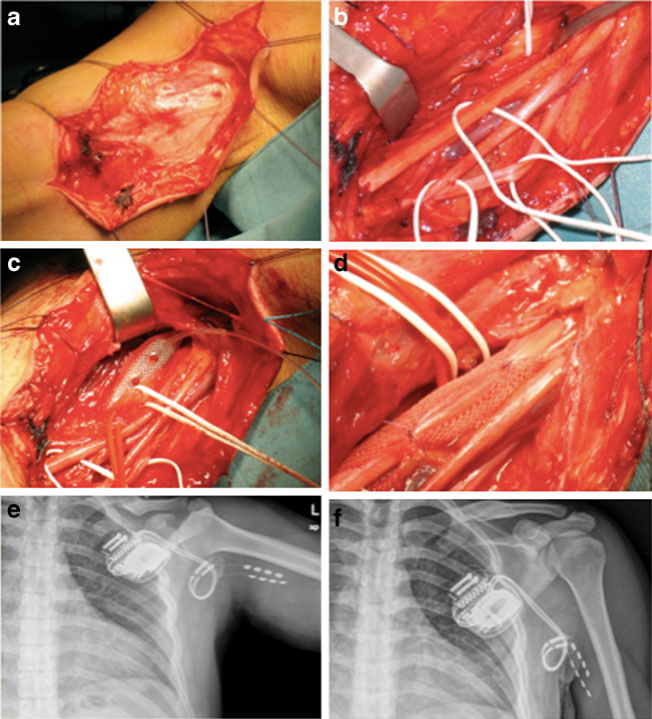
Surgical procedure. The nerves selected for surgery were exposed using the technique of brachial plexus exposure by the axillary approach (**a**–**b**). The leads were placed upon the sensitive portion of the nerve according to Sunderland’s scheme about 4 cm far from the rising nerve, on the lateral aspect of the median nerve (**c**) and carefully anchored by means of lead paddle mesh (**d**) with no absorbable sutures in order to avoid nerve compression. X-ray studies (**e**–**f**) show the position of the leads and IPG with different position of the upper arm

Surgical procedures were performed at the General Hospital of Mestre-Venice between 2007 and 2010. All implantations were performed under general anaesthesia and under strict sterile conditions by one surgeon (G.S.). The nerves selected for surgery were exposed using the technique of brachial plexus exposure by the axillary approach. Patients were supine with the upper arm abducted by 90°, all the nerve and vascular structures were exposed, identified and classified as shown in Fig. 1.

Two on-point leads (Medtronic Inc., Minneapolis) were used for the stimulation of the radial or/and the median nerve, respectively. Each lead has four platinum–iridium electrodes on a silicone-rubber mesh. During the surgical procedure, the leads were placed upon the sensory portion of the nerve according to the Sunderland’s scheme [26] about 7 cm from the nerve origin, on the postero-medial aspect of median nerve and about 5 cm from the antero-medial part of the rising radial nerve and carefully anchored using the paddle lead mesh (Fig. 1C) with no absorbable sutures in order to avoid nerve compression. This technical solution allows nerve protection by the surrounding fibrosis and provides stability of the overall system.

If the patient had motor function preserved at baseline, a stimulation trial was carried out by means of NeuroPulse^TM^ (Bovie Medical Corporation) to confirm the correct position on sensory nerve fibres in order to avoid the stimulation of motor fibres. In three patients (1–3), the on-point leads were placed proximally to the site of lesion; in the other four patients (4–7), the on-point leads were placed distally at the rise of sensitive nerve involved in painful syndrome, after verifying the partial integrity of somatosensory signals. The electrodes were tunnelled under the skin, making several loops to minimize traction or dislocation with the upper arm movement, and then connected to the temporary extensions. During the trial period, an external stimulator (ENS, Medtronic Inc, Minneapolis) was used for a mean of 7 days.

After the trial period, temporary extensions were removed and replaced by permanent extensions which were then connected to an implantable pulse generator (IPG, mod.Prime advanced, Medtronic Inc., Minneapolis) that was secured in the subclavicular subcutaneous pocket.

During the first trial, about 24 h after the procedure, the pulse rate, width and voltage that produced the best response were selected. Stimulator setting was with width and pulse rate fixed for each patient. All patients were set for 24-h stimulation.

### Daily pain evaluation and scales

Patients were requested to evaluate their pain intensity with the use of an 11-point Likert scale (NRS), where “0” indicates “no pain” and “10” indicates “the worst imaginable pain”. Additionally, they were asked to describe their pain with the use of the pain questionnaire [6, 19].

### Pain and clinical follow-up evaluation

Patients were assessed during scheduled follow-up visits for efficacy evaluations after 1 week, 1 month, 6 months and 1 year from the IPG implantation. Evaluation of global pain relief was graded on a percentage scale of four categories: 0–24 % (poor), 25–49 % (fair), 50–74 % (good) and 75–99 % (excellent). Patients underwent NRS scales and pain questionnaires. Finally, to quantify the pain-positive phenomena such as thermal hyperalgesia and allodynia, quantitative sensory testing (QST) was assessed at baseline, 1 month and 12 months.

### Statistics

A two-tailed *t* test was used to compare NRS values and analgesic consumption prior to surgery and during the follow-up. Results at the *P* < 0.05 level were regarded as statistically significant.

## Results

We identified seven patients, all men, fulfilling the inclusion criteria and suitable for PNS. All patients had a post-traumatic brachial plexus lesion or distal peripheral nerve, complaining of severe intractable pain characterized by allodynia, paradoxical pain and ongoing pain. The clinical data are reported in Tables 1 and 2. In four patients, a traumatic peripheral nerve lesion occurred, while in three, a traumatic brachial plexus postganglionic injury caused the peripheral NP syndrome. The patients’ ages ranged from 17 to 68, with a median age of 46. Pain duration prior to PNS ranged from 19 months to 31 years. The mean baseline NRS was 9/10, indicating moderate to severe pain intensity before surgery. The patients described the painful syndrome with several qualities, including cutting sensation, burning sensation and constriction with impressive positive phenomena like electric shock after light touch or movement, several times each day. From the history, all patients had previously received neurosurgical treatments including neurolysis. Therefore, all patients were prescribed conventional medications (analgesics, antidepressants, anticonvulsants) (see Table 2) and repeated nerve blocks. None of the patients met the research diagnostic criteria for complex regional pain syndrome II (CRPS II) [8].

**Table 1:** Summary of clinical data before PNS implantation

Patient no.	Gender, age (years)	Etiology	Duration of plexus or nerve lesion (years, months)	Suggested nerve lesion	Dermatomes or nerve area with abnormal sensation	Site of lesion	Concomitant injury	NRS (at baseline)	Ongoing pain treatment
1	M, 58	Accident at work	8 years	Median, radial nerves	Median, radial nerves	Median and radial nerve lesion at third distal of forearm	Partial amputation of forearm	10	TCA, pregabalin, opioid
2	M, 68	Iatrogenic	19 months	Median nerve	Median nerve	Proximal site of median nerve		8	Pregabalin, opioid
3	M, 42	Accident at work	4 years	Median, radial nerves	Median, radial nerves	Nerve injury at phalaneal level	Distal amputation (II, III, IV fingers)	10	Tramadol, gabapentin
4	M, 47	Accident at work	3 years	Median nerve	Median nerve	C6–C7 lateral cord	Amputation	7	Paracetamol, NSAID, opioid, gabapentin
5	M, 54	Motorcycle accident	31 years	Median nerve	Median nerve	Severe injury at lateral cord		9	–
6	M, 17	Motorcycle accident	32 months	Median nerve	Median nerve	C6 lateral cord		10	High-dosage opioid, pregabalin, TCA
7	M, 36	Motorcycle accident	14 years	Median nerve	Median nerve	(C5)–C6–C7–C8 postganglionic lesion		9	Opioid, pregabalin, TCA

**Table 2:** Painful syndrome phenotype and neuroalgological pattern

Patient no.	Background pain	Spontaneous pain paroxysms			Evoked pain, 0–10 NRS				
	Intensity, NRS	Intensity, NRS	Duration (s)	Frequency	Brush	Pinprick	Repetitive pinprick	Cold	Warm
1	10	7	15	4/day	10	8	5	5	8
2	8	5	30	1/h	9	5	4	3	2
3	10	10	10	1/h	10	1	6	6	9
4	7	8	160	10/h	10	10	10	10	7
5	9	9	5	1/month	7	7	8	5	8
6	10	10	30–45	1/h	5	8	10	7	6
7	9	5	2–3	3/day	6	6	5	5	6

The results based on pain relief were classified as “good” in all patients. The stimulation parameters of PNS were similar in all patients, with a rate pulse of 50 Hz, a width of 250 μs and an amplitude ranging from 0.15 to 0.30 V. This stimulation setting the PNS does not produce any sensation like paresthesia in the skin area.

**Fig. 2:**
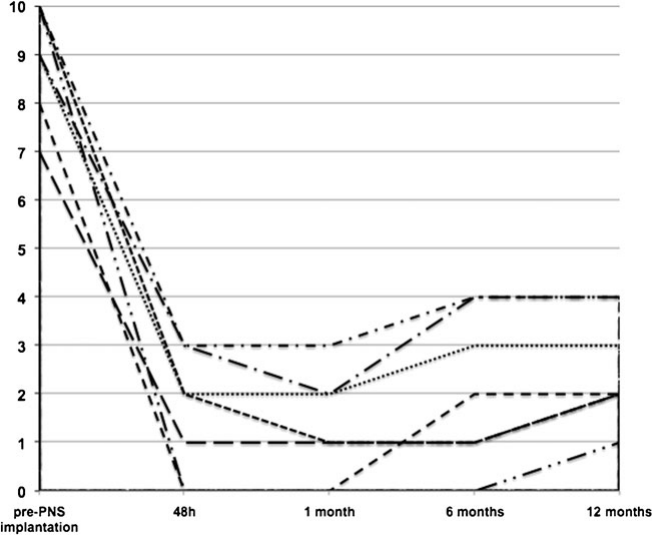
Average pain intensity scores before and after PNS implantation during a follow-up period of 1 year

Overall, pain intensity decreased from an NRS of 9 ± 1.15 before surgery to 2.14 ± 1.57 at the6-month follow-up and to 2.57 ± 1.13 at the 12-month follow-up (*P* < 0.001) (Fig. 2). No complications like infections, dislocation or electrodes dislocations or migrations occurred in any of the patients.

### QST battery at baseline and at follow-up

Thermal thresholds were abnormal in all patients according to the neuroanatomical distribution of negative signs as expected by the sensory clinical evaluation; in particular, we found higher cold and warm sensation thresholds in selective skin areas. In six of seven patients at baseline, we found positive phenomena characterized by cold or warm allodynia and in five wrong painful sensation after cold and warm stimuli into the non-painful range. The abnormal painful sensation was reported as an electric shock or burning sensation with wide skin area distribution, higher than expected by the nerve injured distribution, suggesting peripheral or central hypersensitivity phenomena.

After 6 months, at the follow-up evaluation, thermal QST evaluation showed unchanged negative sign pattern and attenuation or complete resolution of positive phenomena (Fig. 2).

## Discussion

Peripheral nerve stimulation (PNS) is a neuro-modulation technique in which electrical current is applied to the peripheral nerves to improve chronic pain. It was first described by Wall and Sweet in 1967 [30]; they used an electrode to stimulate a peripheral nerve in post-traumatic neuralgic pain. A variety of techniques have since been developed [20]. In the 1970s, PNS was seldom performed because the high morbidity and poor long-term outcomes related to poor patient selection and technical limitations for inadequate devices. However, growing evidences suggested that PNS is effective, in particular NP syndromes characterized by peripheral nerve lesions or irritation with pain or positive phenomena with a localized peripheral nerve distribution [9, 21, 27, 31, 32].

In this study, we show a selected case series of patients complaining of severe neuropathic pain involving the upper arm with pain phenomena extremely consistent to a peripheral nerve distribution. This clinical picture is usually at high risk for non-responsiveness with other neuro-modulation techniques such as spinal cord stimulation (SCS) or motor cortex stimulation (MCS) [1, 3, 20]. In particular, SCS is reported to be more effective in painful syndrome with spinal hyperactivity in the dorsal part of spinal cord but compared with PNS is less selective in covering the painful skin area of peripheral nerve injuries and has higher risk of dislocation [13, 26]. MCS in peripheral neuropathic pain has been applied in few case series [15] and has unpredictable antalgic effect to be compared with the other techniques.

PNS pain relief can be explained by the “gate-control” theory of pain [16]; the peripheral nerve stimulation produces direct electrical effects by recruitment of primary afferent A-beta and A-delta fibres that project at the spinothalamic tract and dorsal columns and A-alpha fibres that cause segmental inhibition through presynaptic inhibitory interneurons. These electrical effects seem to be more selective and effective than SCS.

However, peripheral nerve stimulation through a percutaneous approach [11, 17]—in particular, for upper and lower limb—has a less favourable chronic outcome and higher morbidity due to displacement-related movement of adjacent structures (i.e. tendons, nerves, vascular structures). These dynamic forces can affect distraction and translation of the electrodes from their associated nerves. Otherwise, for similar reasons, the migration of electrodes is one of the most frequent complications, occurring in up to 33 % of cases [12], and requires revision. For these reasons, we propose this new surgical technique with the allocation of on-point lead into soft tissue of the armpit, without tendon–muscular structure, which avoids the nerve compression [26]. Notably, this technique minimizes the chance of electrode dislocation, is selective on the target nerve branches and allows a low-intensity stimulation that saves battery life.

In our opinion, the success of PNS implantation in all our cases is based on a careful patient selection process. We applied selection criteria based on literature review [9, 26] and our clinical and neurophysiological experience in neuropathic pain studies (the details of which are included in the paragraph on Methods). We improve the sensory profile evaluation using a semiquantitative instrument such as a quantitative sensory testing battery, which is particularly useful for quantifying positive and negative signs at baseline and after treatment [2].

In literature, the use of a prognostic nerve anaesthetic block prior to PNS has been suggested by several groups [13, 29], and from common practice it is well accepted that failure to respond to a nerve block is a poor prognostic factor for PNS. For that reason, complete although temporary *pain relief* following a diagnostic nerve block with local anaesthetics was required as a pre-test in our screening evaluation. The use of the local anaesthetic injection may help us to confirm the selection of the nerve target and identify the prevailing peripheral source of the particular pain syndrome.

As reported also by other groups [29], we observe that PNS modified neither heat nor cold thresholds assessed by the QST battery. Therefore, the main finding for the QST battery was the clear anti-allodynic or anti-hyperalgesic effect. The improvement of positive phenomena is a direct effect of the PNS stimulation, while the negative signs in our patients are the consequence of the nerve lesion, and PNS is obviously ineffective in improving a loss of peripheral nerve function such as sensory sensation. However, PNS can affect changes in nociceptive signalling that modulate peripheral and central sensitization leading to a reduction of allodynia.

The 1-year follow-up confirms the long-lasting beneficial effect of SCS, nearly unchanged between visit after 1 month and visit after 12 months.

Finally, we assessed the usefulness of thermal QST to detect the positive phenomena in our patients, with a sort of stereotypical and reliable QST pattern also confirmed by several observations (data not shown) and, above all, for follow-up evaluation to clearly detect the anti-allodynic effect of PNS.

## Conclusions

This new PNS technique of electrode implantation can result in significant pain relief in carefully selected patients with peripheral neuropathic pain due to post-traumatic nerve injuries, reducing the complication rate and preserved neuroanatomical structures. It should therefore be considered as a reasonable treatment of patients suffering from otherwise intractable painful neuropathies of the upper arm.
